# Association Between Different Indicators of Obesity and Depression in Adults in Qingdao, China: A Cross-Sectional Study

**DOI:** 10.3389/fendo.2018.00549

**Published:** 2018-10-10

**Authors:** Jing Cui, Xiufen Sun, Xiaojing Li, Ma Ke, Jianping Sun, Nafeesa Yasmeen, Jamal Muhammad Khan, Hualei Xin, Shouyong Xue, Zulqarnain Baloch

**Affiliations:** ^1^Qingdao Municipal Center for Disease Control and Prevention, Qingdao, China; ^2^Qingdao Institute of Preventive Medicine, Qingdao, China; ^3^Qingdao Shi'nan Municipal Center for Disease Control and Prevention, Qingdao, China; ^4^College of Traditional Chinese Medicine, Shandong University of Traditional Chinese Medicine, Jinan, China; ^5^Institute of Microbiology, Agriculture University Faisalabad Pakistan, Bahawalpur, Pakistan; ^6^Department of Patho-biology, The Islamia University of Bahawalpur, University College of Veterinary and Animal Sciences, Bahawalpur, Pakistan; ^7^Qingdao Shi'bei Municipal Center for Disease Control and Prevention, Qingdao, China; ^8^College of Veterinary Medicine, South China Agricultural University, Guangzhou, China

**Keywords:** depression, body mass index, waist circumference, waist-to-hip ratio, cross-sectional study

## Abstract

**Background:** This study was designed to investigate the perceived relationship between body weight and depression risk in a Chinese population in Qingdao, China.

**Methods:** A population-based cross-sectional survey was performed with 4,573 participants (between 35 and 74 years) from the year 2009 to 2012 in Qingdao, China. We applied the Zung self-rating depression scale to ascertain the level of depression in participants. The associations between different indicators of obesity [body mass index (BMI), waist circumference (WC), and waist-to-hip ratio (WHR)] and depression were assessed by logistic regression based on the Chinese criteria of obesity. Sensitivity analysis was done based on the Asian and WHO criteria of obesity.

**Results:** The Zung scores for the 243 participants (5.2%) were over 45 and they were entitled as depression. Furthermore, multivariable logistic analyses revealed that being overweight [odds ratios (OR): 1.48, 95% confidence intervals [95% CI]: 1.08–2.03] and having abdominal obesity (WC category in Chinese criteria) (OR: 1.47, 95% CI: 1.08-2.00) were often associated with a higher risk for depression compared to normal weight subjects. Sensitivity analysis revealed that abdominal obesity (Asian criterion) (OR: 1.41, 95% CI: 1.03-1.91) was a significant risk factor for depression. Similarly, being overweight (WHO criterion) (OR: 1.39, 95% CI: 1.03-1.87) was an obvious risk factor for depression.

**Conclusion:** Being overweight and having abdominal obesity (WC category) were found to be linked with a higher risk of depression. However, abdominal obesity (WHR category) was not associated with depression.

## Introduction

Significant progress has indeed been made in improving public health in the world; however, many serious health issues still need to be resolved such as depression and obesity. The global prevalence of being overweight and obese was estimated to be 39% and 13% in adults aged 18 years and over in 2017, respectively (1). In 2018, the World Health Organization (WHO) estimates that depression is seen to affect approximately 300 million people worldwide (2). In China, the prevalence of obesity has been continuously increasing during the last few decades (3–5). Additionally, depression is also frequently diagnosed at public and private clinics. According to health officials, approximately 26 million people are struggling with symptoms of mild depression annually in China (6).

Several population-based studies conducted in Western countries demonstrated that the risk for depression was positively associated with being overweight and obesity (7–12). In another study, a U-shaped association between BMI and depression has been reported (10). However, estimates obtained from the Chinese population are found to be controversial (13–15). Several research activities have reported a negative correlation between the risk for depression and obesity among middle-aged and the elderly Chinese (13, 14). In contrast, Li et al. observed a significant positive association between obesity and increased depression scores in the Chinese elderly (aged ≥ 65 years) (15). Nonetheless, mechanisms underlying the association between obesity and depression are still unclear. Some studies have shown that the brain–reward regions for depression and obesity, such as the dopaminergic pathways, can enhance positive mood. Yet, they also cause increased intake of “comfort” food, which may lead to obesity (16, 17). As a matter of fact, the perceptions regarding obesity are different between the Western world and the Chinese. While obesity is a stigma in Western countries (18), it is considered as a symbol of wealth in China (13–15). Regardless of these differences, it may be due to population diversity, different body weight criteria, and depression standards in China.

The association between obesity and depression has been normally studied in adolescents and the elderly population in China (19–21); yet there is a need to explore various indicators of obesity in detail. Hence, this large population-based cross-sectional survey was carried out to assess the potential relationship between depression and different body weight levels among the general population of Qingdao, China.

## Methods

### Ethical considerations

All the participants voluntarily signed the consent prior to their participation. The security, anonymity, and the privacy of participants were strictly respected. This study was approved by the Ethics Committee of the Qingdao Municipal Center for Disease Control and Prevention.

### Study population

This cross-sectional population-based survey was conducted in the eastern city of Qingdao, Mainland China during 2009 to 2012. Inclusion and exclusion criteria have already been described in our previous study (22). Briefly, a total of 6,100 Chinese adults (between 35-74 years old) were selected with a stratified, random cluster sampling procedure and invited to participate in the survey. A total of 5,110 individuals agreed to take part in our survey, with a response rate of 83.8%. From the 5,110 individuals, 454 participants were excluded due to an insufficient Zung score or body mass index (BMI) or waist circumference (WC) or waist-to-hip ratio (WHR) baseline information. Therefore, results from the current study were based on data from 4,656 participants, accounting for 76.3% of the initially invited individuals.

### Questionnaires

A standard questionnaire was designed to collect basic information which included age, gender, marital status, educational background, occupation, smoking, and alcohol consumption. The marital status was divided into married/cohabiting and unmarried (single, divorced, or widowed). Educational attainment was categorized into three groups: illiterate/ elementary school, junior high school, and senior high school or higher. Occupational physical activity (PA) was categorized into light (housewife, retired, or unemployed), moderate (teacher, doctor, or nurse), and heavy (worker, farmer, or fisherman), according to occupation. Smoking was classified into current smokers (smoking every day and occasional smoking in the past years) and noncurrent smokers (including ex-smokers and nonsmokers). Alcohol consumption was categorized into regular drinkers (drinking every day in the past years) and noncurrent drinkers (including ex-drinkers, rare drinkers, and nondrinkers). Personal monthly income was categorized into ≤ 999 Chinese Yuan (CNY), 1,000–2,999 CNY, and ≥3,000 CNY.

The plasma glucose level was determined by the glucose oxidase method. All subjects (without prediagnosed diabetes) underwent a standard 2-h 75-g oral glucose tolerance test. Diabetes was diagnosed on the basis of the WHO/International Diabetes Federation criteria (23). Subjects with fasting plasma glucose (FPG) level of ≥7.0 mmol/l and/or 2-h postload plasma glucose (2-hPG) level of ≥11.0 mmol/l were diagnosed with diabetes. The individual was taken as nondiabetic if FPG was <7.0 mmol/l and/or 2-hPG <11.1 mmol/l.

Blood pressure was measured using a mercury sphygmomanometer. Measurements were taken three times in 5-min intervals. The mean of the three readings was used for data analysis. Hypertension was defined by a mean systolic blood pressure of ≥140 mmHg and/or a mean diastolic blood pressure of ≥90 mmHg and/or an established diagnosis of hypertension at the baseline (24).

### Anthropometric measurement

We measured the participant's height and weight while wearing light clothes and no shoes. The BMI was calculated as the weight in kilograms divided by the height in squared meters (kg/m^2^). Waist circumference (WC) was measured at the midpoint between the lower rib margin and the iliac crest. Hip circumference (HC) was measured at the maximal horizontal girth between the waist and the thigh. The WHR was calculated by dividing WC (in cm) by HC (cm).

### Assessment of depression

Depression was assessed using the Zung Depression Rating Scale (ZDRS) (25, 26). The ZDRS questionnaire contains 20 questions divided into 10 positively and 10 negatively phrased questions. Each question was scored as 1 through 4, and the total scores ranged from 20 to 80 based on the participant's feelings (sadness, indifference, or happiness) toward family, work, and living conditions, respectively. Participants were divided into two major groups: normal (20–44) and depressed (≥45) (25).

### Criteria for obesity

Overweight and obesity were defined in accordance with the published criteria (26, 27). According to the Chinese criteria, subjects were divided into four BMI categorical groups as: underweight (BMI < 18.5 kg/m^2^), normal weight (18.5–23.9 kg/m^2^), overweight (24.0–27.9 kg/m^2^), and obese (≥28.0 kg/m^2^). According to Asian criteria, subjects were subdivided as: underweight (BMI < 18.5 kg/m^2^), normal weight (18.5–22.9 kg/m^2^), overweight (23.0–24.9 kg/m^2^), and obese (≥25.0 kg/m^2^) (27) and according to the WHO criteria as: underweight (BMI < 18.5 kg/m^2^), normal weight (18.5–24.9 kg/m^2^), overweight (25.0–29.9 kg/m^2^), and obese (≥30.0 kg/m^2^) (28).

Based on the value of WC, subjects were further subdivided into two groups as follows: normal (<85.0 cm for males and <80.0 cm for females); abdominal obesity, (≥85.0 cm for males and ≥80.0 cm for females) according to Chinese criteria (26). Alternatively, subjects were subdivided into: normal (<90.0 cm for males and <80.0 cm for females) and abdominal obesity, (≥90.0 cm for males and ≥ 80.0 cm for females) according to the Asian guidelines (27). Finally, according to the WHO guidelines, subjects were divided into normal (<94.0 cm for males and <80.0 cm for females) and abdominal obesity (≥94.0 cm for males and ≥80.0 cm for females) (28).

Subjects were divided into two categories based on their WHR cutoffs points (Chinese guidelines): normal (<0.9 for males and <0.85 for females) and abdominal obesity (≥0.90 for males and ≥ 0.85 for females) (26); or the Asian WHR cutoff points: normal (<0.95 for males and <0.80 for females) and abdominal obesity (≥0.95 for males and ≥0.80 for females) (29) or the WHO WHR cutoff points: normal (<1.00 for males and <0.85 for females) and abdominal obesity (≥1.00 for males and ≥0.85 for females) (28).

### Statistical analysis

Statistical analyses were performed using IBM SPSS Statistics 17.0. Continuous variables and categorical variables were presented as the mean ± standard deviation and the number (percentages), respectively. Using data from the 2010 census in Qingdao, the age-standardized prevalence of depression was calculated according to different obesity criterion for the age group of 35–74 years. Logistic regression was used to determine the possible link between the risk for depression and different indicators of obesity (BMI, WC, and WHR). Odds ratios (ORs) and 95% confidence intervals (CIs) were estimated by logistic regression to obtain the association between depression and each variable. Sensitivity analysis was done based on the Asian and WHO criteria of obesity. A *P-*value <0.05 was considered to be statistically significant.

## Results

The demographic information, social-economic status, lifestyle information, and anthropometric measurement have been shown in Table 1. In this study, a total of 4,656 participants were enrolled; among them, 1,804 were males (38.6%) and 2,852 were females (61.4%). The mean age was 52.3 ± 10.7 years (rang 35–74 years).

**Table 1:** Characteristics of the population included in the study.

**Characteristic**	**Total *N* = 4,656**
Sex, female, *n* (%)	2852 (61.3)
Age, years (mean ± SD)	52.3 ± 10.7
Zung score (mean ± SD)	29.8 ± 7.9
Depression, *n* (%)	243 (5.2)
BMI (kg/m2, mean ± SD)?	25.0 ± 3.7
Normal*, n* (%)	1871 (40.2)
Underweight, *n* (%)	83 (1.8)
Overweight, *n* (%)	1815 (39.0)
Obese, *n* (%)	887 (19.1)
WC (cm, mean ± SD)?	83.8 ± 10.6
Normal, *n* (%)	1982 (42.6)
Abdominal obesity, *n* (%)	2674 (57.4)
WHR (mean ± SD) ?	0.87 ± 0.07
Normal, *n* (%)	2306 (49.5)
Abdominal obesity, *n* (%)	2350 (50.5)
Hypertension, *n* (%)	2159 (46.5)
Diabetes, *n* (%)	741 (15.9)
Urban living, *n* (%)	1384 (29.7)
Married, *n* (%)	4361 (93.7)
**EDUCATIONAL ATTAINMENT**, ***n*** **(%)**	
Illiterate /elementary school	1904 (41.0)
Junior high school	1723 (37.1)
Senior high school or higher	1022 (22.0)
**OCCUPATIONAL PA**, ***n*** **(%)**	
Light	483 (10.5)
Moderate	346 (7.5)
Heavy	3796 (82.1)
Current smoker*, n* (%)	1137 (24.8)
Current drinker*, n* (%)	751 (16.1)
**INCOME (CNY/MONTH)**, ***n*** **(%)**	
≤599	2372 (52.4)
600–1999	1820 (40.2)
≥2000	332 (7.4)

*BMI, body mass index; WC, waist circumference; HC, hip circumference; WHR, waist-to-hip ratio; PA, physical activity; CNY, Chinese Yuan. ? obesity and abdominal obesity is defined according to Chinese criteria. n, number*.

**Figure 1:**
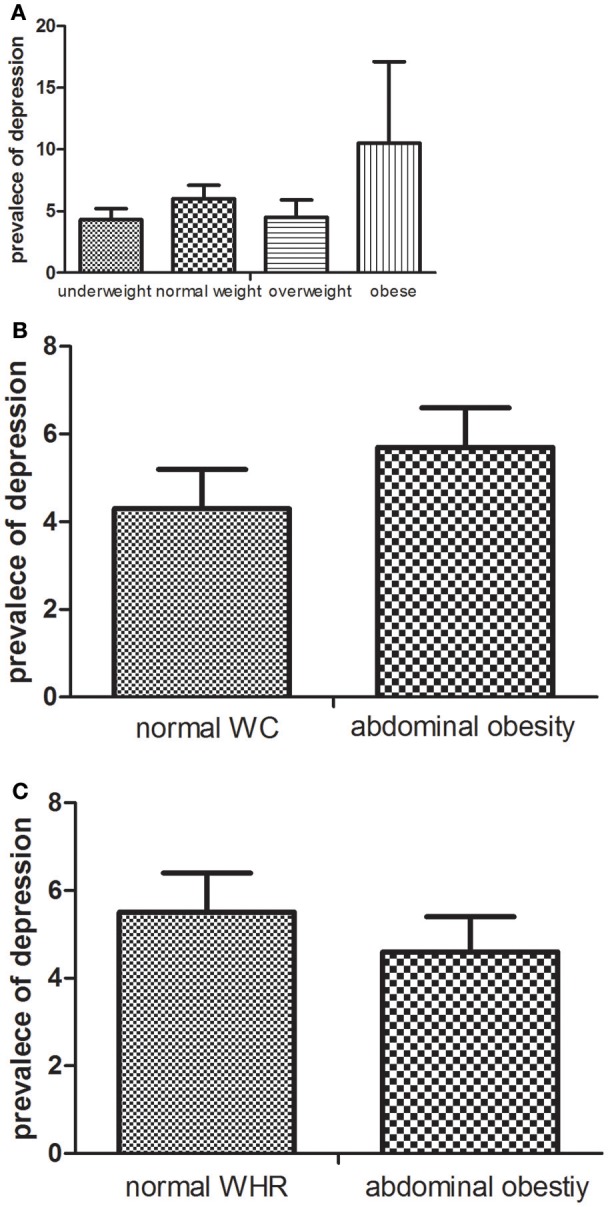
The age-standardized prevalence (%) of depression according to BMI **(A)**, WC **(B)**, and WHR **(C)**.

According to the BMI category, the age-standardized prevalence of depression was higher in underweight (10.5%) and overweight participants (6.0%) compared with the normal weight (4.3%); however, the difference was not statistically significant. Based on the WC category, a slightly higher age-standardized prevalence of depression was observed in the abdominal obesity participants (5.7%) compared with the normal WC participants (4.3%), and the difference was statistically significant. The corresponding age-standardized prevalence of depression was 5.5% in normal WHR participants and 4.7% in the abdominal obesity participants, respectively, and the difference was nonsignificant. The age-standardized prevalence of depression according to BMI, WC, and WHR has shown in Figure 1.

In this study, we determined the role of potential risk factors in depression using multivariable regression analysis according to the Chinese criterion (Table 2). Overweight indicated higher risk of depression (OR: 1.49, 95%CI: 1.08-2.05) compared with normal weight. We observed a significant relationship between abdominal obesity and depression risk according to WC.

**Table 2:** Risk factors associated with depression determined by multivariable logistic regression according to the Chinese criterion.

	**Model 1**	**Model 2**	**Model 3**
Female	1.09 (0.72–1.62)	1.05 (0.70–1.56)	1.09 (0.73–1.62)
age	1.01 (1.00–1.03)	1.01 (1.00–1.03)	1.01 (1.00–1.03)
**BMI CATEGORY**			
Normal	1.00		
Underweight	1.24 (0.44–3.50)		
Overweight	1.48 (1.08–2.03)^*^;		
Obese	1.16 (0.76–1.78)		
**WC CATEGORY**			
Abdominal obesity		**1.47 (1.08**–2.00)^*^;	
**WHR CATEGORY**			
Abdominal obesity			0.86 (0.64–1.17)
Hypertension	0.81 (0.60–1.10)	0.78 (0.58–1.06)	0.86 (0.64–1.17)
Diabetes	0.81 (0.55–1.21)	0.80 (0.54–1.19)	0.84 (0.56–1.24)
Urban living	1.45 (0.95–2.22)	1.44 (0.95–2.20)	1.49 (0.98–2.28)
Unmarried	**1.96 (1.26**–3.06)^*^	**1.93 (1.24**–3.01)^*^	**1.93 (1.24**–3.01)^*^
**EDUCATIONAL ATTAINMENT**			
Illiterate /elementary school	1.00	1.00	1.00
Junior high school	0.81 (0.55–1.19)	0.79 (0.54–1.16)	0.79 (0.54–1.17)
Senior high school or higher	0.69 (0.41–1.14)	0.68 (0.41–1.13)	0.66 (0.40–1.11)
**OCCUPATIONAL PHYSICAL ACTIVITY**			
Light	1.00	1.00	1.00
Moderate	0.88 (0.48–1.61)	0.86 (0.47–1.56)	0.90 (0.49–1.64)
Heavy	0.71 (0.46–1.11)	0.72 (0.46–1.12)	0.72 (0.46–1.12)
Current smoker	0.76 (0.51–1.15)	0.77 (0.51–1.16)	0.79 (0.52–1.19)
Current drinker	0.85 (0.55–1.31)	0.84 (0.55–1.30)	0.86 (0.56–1.32)
**INCOME (CHINESE YUAN/MONTH)**			
≤599	1.00	1.00	1.00
600–1999	0.91 (0.49–1.71)	0.92 (0.49–1.71)	0.91 (0.48–1.70)
≥2000	1.14 (0.65–2.00)	1.14 (0.65–2.00)	1.12 (0.64–1.98)

*Model 1, model 2 and Model 3, the risk factors listed in the table were entered at the same time*.

* *P < 0.05 for factors associated with depression*.

Next, we performed a sensitivity analysis according to the Asian and WHO criteria (model 1 and model 2, respectively; Table 3). In model 1, sensitivity analysis based on Asian criterion revealed that overweight and obesity (BMI category) were not significantly associated with depression, while abdominal obesity (according to WC) was significantly associated with depression similar to that of Chinese criterion. In model 2, based on the WHO criterion, abdominal obesity (according to WC) was not significantly associated with depression, while overweight participants in the BMI category were significantly associated with depression.

**Table 3:** Sensitivity analyses for depression according to the Asian and WHO criteria.

	**Model 1**	**Model 2**
**BMI CATEGORY**		
Normal	1.00	1.00
Underweight	1.20 (0.42–3.42)	1.14 (0.41–3.21)
Overweight	1.14 (0.75–1.71)	**1.39 (1.03**–1.87)^*^
Obese	1.33 (0.95–1.88)	0.56 (0.28–1.13)
**WC CATEGORY**		
Abdominal obesity	**1.41 (1.03**–1.91)^*^	1.32 (0.96–1.82)
**WHR CATEGORY**		
Abdominal obesity	1.19 (0.83–1.70)	1.02 (0.72–1.46)

*Model 1 is according to Asia criterion. Model 2 is according to WHO criterion. All models were adjusted for gender, age, hypertension, diabetes, resident districts, marital status, educational attainment, occupational physical activity, smoking status, alcohol drinking status, personal monthly income. BMI category, WC category, and WHR category entered models separately*.

* *P < 0.05 for factors associated with depression*.

## Discussion

In this study, we investigated the association between depression and body weight among adult people in Qingdao, China. Our results showed that the prevalence of depression was higher among the overweight participants (according to BMI category in China) and abdominal obesity participants (according to WC category in China) compared with other groups. Additionally, we defined prevalence of depression by using a Zung score equal to or greater than 45. In Qingdao, the overall prevalence of depression rate of 5.2% (5.5% in males and 5.0% in females) was lower than the documented rates in South Korea (overall 5.7%, 3.9% in males and 7.0% in females) (30). Additionally, a cross-sectional study of 512 891 Chinese adults, aged 30–79 years, had also revealed a lower pooled prevalence of depression compared with the current study (2.4%) (31). This difference might be due to social and family factors. Residents of the eastern coastal areas could not convincingly adapt to rapid industrialization and urbanization; thus it lead to depression. Secondly, accompanied with China entering the aging society, the “empty nest” phenomenon is the leading cause of unaccompanied, unattended, and psychosocial problems (such as depression) in middle-aged and elderly adults (32, 33). Depression, in turn, affects the middle-aged and the elderly population's social and physical activities, which strongly influenced the wellbeing of elderly adults (34).

The prevalence of depression is higher in underweight than normal individuals. However, there was an insignificant positive association between underweight and depression in general adults in Mainland China. Our results are inconsistent with the estimates reported in Japan, Korea, and Taiwan (30, 13, 35, 36). These trends can be observed in different age populations, and underweight elderly adults are far more likely to be depressed (30, 13, 35, 36). Yet, the common population tends to have a distortion of body weight, which entitles thinness as a beauty symbol owing to social standard (37). Our observations also support this fact.

The prevalence of depression was higher in overweight and abdominal obesity (WC category) participants compared with the normal participants. In contrast, we did not observe a similar association in obese and abdominal obesity (WHR category) participants. This trend can be explained by the Chinese cultural heritage that usually associates overweight with a higher economic status, since those who can afford to eat more could attain more body weight and vice versa. In China, prevalence of obesity was substantially higher in rich people (38, 39). The overweight carries a social stigma, such as body image, self-esteem, and social life, which can help a bit for depression (40).

In this study, our analysis revealed a significant positive association between being overweight and depression, while a nonsignificant association between being obese and depression. However, the association of BMI with depression is still controversial. A nonsignificant positive relationship between being overweight and depression has been reported in developed countries such as Canada (41). Additionally, Palinkas et al. observed a nonsignificant inverse association between being overweight and depression in women (42). Moreover, a nonsignificant negative association between depression and obesity has also been reported previously (12, 43). In contrast, several studies reported a significant positive association between depression and obesity (11, 12, 20, 21, 44, 45). Young people regarded thinness as a beauty symbol. However, middle-aged and elderly people are still more likely to view obesity as a symbol of wealth and happiness in the traditional Chinese culture. Meanwhile, in this study, participants are mainly middle-aged and the elderly people aged 35 years or over, which is the possible reason for the current observations.

Next, we examined the effect of abdominal obesity on depression. We observed a significant positive association between abdominal obesity and depression. These results are in agreement with those of Takeuchi et al. (46) and Vogelzangs et al. (47). In contrast, Herva et al. (48), Gil et al. (49), and Zavala GA (12) reported a nonsignificant positive association between depression and abdominal obesity. The absence of significant associations between abdominal obesity (WHR category) and depression were inconsistent with the results reported by Ahlberg et al. (50).

Here, for the first time, we reported a significant association between being overweight, abdominal obesity (WC category), and depression in the general population of Qingdao China, but not in obese or abdominal obesity (WHR category) participants, which might be due to the following reasons. First, cultural factors are known to influence the association between depression and body weight. A higher socioeconomic status is generally associated with more concern for body image and abnormal body weight, which can possibly be a stress factor that leads to depression (51, 52). Second, diet is an important factor in the association between depression and body weight status. Emotions of individuals can also affect food intake. Depression was associated with emotional eating such as negative emotional overeaters (53). Moreover, a negative emotion, such as depression, is further inclined to uncontrolled eating and/or overeating (54). However, this will ultimately lead to a high body weight gain. Finally, we cannot underestimate the roles of neurological mechanisms (55, 17, 16) and/or genetic susceptibility (56), which may also lead to depression and an abnormal weight status.

Previous studies had reported conflicting results in China, partly because different studies had used different criteria to define obesity. Therefore, in this study, we performed sensitivity analyses according to the Asian and WHO criteria of obesity. Sensitivity analyses showed a significant positive association between depression and abdominal obesity (WC category in Asian criteria), and being overweight (BMI category in WHO criteria) after adjustment for gender, age, hypertension, diabetes, resident districts, marital status, educational attainment, occupational PA, smoking status, alcohol-drinking status, and personal monthly income. These results were slightly different from the results produced using the Chinese criteria. Taken together, we suggest that the association between depression and weight status is influenced by the criteria adopted. Additionally, the reciprocal and complex associations of being overweight, abdominal obesity, and depression are strongly intertwined, and these phenomena are likely to be true for the earlier-mentioned results. Hence, further large-scale research is needed to reveal the association between obesity and depression among the whole Chinese population.

This study suffered from a few limitations. First, the present study was a cross-sectional study that does not reflect the underlying mechanisms between depression and weight status. Therefore, a follow-up study is imperative. Second, this study was performed on a relatively small sample size of the adult community in Qingdao, China.

## Conclusion

We observed that overweight and abdominal obesity (WC category) participants were at a higher risk of depression according to Chinese criterion. However, abdominal obesity according to WHR was not associated with depression. Furthermore, Asian and WHO criteria of obesity might influence the association between depression and body weight status. On all accounts, controlling being overweight and having abdominal obesity had a protective impact on depression. Future research will involve a larger multicenter study in China to further investigate the relationship between body weight and depression.

## Ethics and consent to participate

All the participants voluntarily signed the informed consent before their participation and the consent of ethics, including three urban districts (Shinan, Shibei, and Sifang) and three rural districts (Huangdao, Jiaonan, and Jimo), was obtained from the ethics committee in Qingdao Municipal Center for Disease Control and Prevention. This study was approved by the local ethics committee at Qingdao Municipal Health and Family planning commission.

## Availability of data and materials

The aggregate data supporting findings contained within this manuscript will be shared upon request submitted by the corresponding author. Identifying patient data will not be shared.

## Author contributions

ZB, JC, and JS were the primary authors and leading investigators. NY, JC, MK, ZB, and HX carried out the experiments, analyzed experimental results. ZB, JC, and JS wrote the manuscript. All authors read and approved the final manuscript.

### Conflict of interest statement

The authors declare that the research was conducted in the absence of any commercial or financial relationships that could be construed as a potential conflict of interest.

## Abbreviations

BMI: Body mass index
CI: Confidence intervalsl
OR: Odds ratio
WC: waist circumference
WHO: World Health Organisation.
